# Is There Any Correlation between Migraine Attacks and Iron Deficiency Anemia? A Case-Control Study

**Published:** 2019-07-01

**Authors:** Ali Tayyebi, Maryam Poursadeghfard, Masoumeh Nazeri, Tahereh Pousadeghfard

**Affiliations:** 1 Medical Student, Shiraz University of Medical Sciences, Shiraz, Iran; 2 Clinical Neurology Research Center, Shiraz University of Medical Sciences, Shiraz, Iran; 3 Department of Mathematics and Statistics, Firoozabad Branch, Islamic Azad University, Firoozabad, Iran

**Keywords:** Migraine, Iron-deficiency anemia, Correlation, Relation

## Abstract

**Background: **Migraine headache is an episodic abnormality which usually presents with a severe headache, accompanied by nausea, photo and sound sensitivity, and autonomic symptoms. Iron accumulation in brain, especially peri-aqueductal grey is associated with duration of the disease, and apparently there is an association between body iron storage status and the incidence of migraine; hence, the present study was conducted to investigate the plausible association between iron-deficiency anemia and migraine in a case-control design.

**Materials and Methods: **After signing the written informed consent, the blood samples were collected by a well-trained technician from the patients proved to have migraine, those having migraine clinical criteria and those having migraine attack frequency as high as that prophylaxis was required, and non-migraine healthy individuals, those having not migraine and anemia except iron-deficiency anemia. Based on the sample size, each group composed of samples with at least 100 individuals.

**Results: **There were statistically significant differences between female cases and controls regarding hemoglobin, serum ferritin levels and iron-deficiency anemia (P: .0004; .006; .001), but no differences were observed among males (P: .606; .38; .303). Furthermore, the case-control comparisons revealed a significant difference in iron-deficiency anemia (P: .032), but no significant difference was seen in hemoglobin and serum ferritin levels (P: .161; .178).

**Conclusion: **The present study suggests an association between iron-deficiency anemia, hemoglobin and serum ferritin levels and the incidence of migraine in females. As a result, there might be an association between body iron storage status and the incidence of migraine, especially among females, reflecting the fact that iron supplements might be an effective treatment or prophylaxis in patients with migraine associated with iron-deficiency anemia. However, further studies are required to provide a conclusive answer to the issues remained controversial.

## Introduction

 Migraine headache is a periodic disorder, the most important of which is usually a severe headache associated with nausea or a sensitivity to sound and light, and is often accompanied by symptoms in the autonomic nervous system. This disease is one of the most common causes of referral to neurology clinics^1^. Iron is the most commonly received nutrient in human diet. Iron deficiency affects many of the cellular functions and processes such as oxygen transmission, neuronal malignancies, electron storage and transport, oxidative phosphorylation, neurotransmitter metabolism, immune function, and DNA synthesis^2^. 

Studies have indicated that iron plays a major role in the synthesis of serotonin, dopamine and norepinephrine. Meanwhile, the serotonin level of the brain as a mediator decreases in migraine headaches^   3 ^. Serotonin is a key neurotransmitter in migraine neurobiology and its relationship with migraine has been shown. These studies have indicated that the serotonin level in migraine attacks decreases in the central nervous system and increases in the peripheral nervous system^   4 ^. Iron Deficiency Anemia (IDA) may in some way lead to a reduction in serotonin^   5 ^. Many data declared that metabolic abnormalities in the brain following iron deficiency anemia lead to a reduction in neuronal activities. Also,according to some surveys, the level of monoamine oxidase enzyme activity reduces in both migraine and in IDA^   6 ^.

In addition, both migraine and iron-deficiency anemia are more common in young women, although there are very few studies exist about the relationship between these two diseases^        2 ^.

So, if the relationship between iron-deficiency anemia and migraine attacksis confirmed with more evidence, the treatment of iron IDA can greatly influence the rate of migraine attacks and the life quality of migraine patients. Therefore, due to the importance of the above-mentioned and unknown mechanism of migraine, and also due toless studies on the relationship between IDA and migraine, the present study was conducted to determine the relationship between these two diseases.

## MATERIALS AND METHODS

 The study was a cross-sectional case-control study that was conducted at Imam Reza clinic, Shiraz University of Medical Sciences from February 2017 to June 2017.

After obtaining the written consent form, the subjects were selected through available sampling. *patients* having International Headache Society (IHS)-based migraine criteria^   7 ^ and the prevalence of their migraine attacks was as high or severe as requiring prophylaxis met the inclusion criteria of the study. All patients were examined and selected by a neurologist. The patients were excluded from the study due to the following conditions:

1. Having had iron supplementation in the last six months.
2. Having a confirmed anemia, with the exception of iron-deficiency anemia such as thalassemia.
3. Havingchronic illnesssuch as the asthma and chronic renal failure.

The control group consisted of healthy subjects without migraine who had referred to the laboratory for a periodic laboratory review only. The following people were also excluded from the control group:

1. People with a history of confirmed migraine and anemia, with the exception of iron- deficiency anemia.
2. Patients in the acute phase of inflammatory or infectious disease.

Iron-deficiencyanemia is defined asa decrease inthe amount of hemoglobin (<12 mg/dl for women and <16 mg/dl for men) and ferritin level (<50 ng/dl) for each sex.

This research wasapproved by the Ethics Committee of Shiraz University of Medical Sciences(Registration No: 93-01-01-8823).

**Statistical analysis**

To describe the data, mean, standard deviation and relative prevalence were used. The comparison between the two groups was performed using Chi-square and independent T-test. Data analysis was performed using SPSS 18. A value of P< 0.05 was considered statistically significant.

## Results

 In this study, the case group consisted of 100 patients with an average age of 37.63 ± 9.93years, which included 76 women with an average age of 37.79 ± 10.28 years and 24 men with an average age of 37.13 ± 8.93 years. The control group included 100 normal subjects with an average age of 34.93 ± 12.25 years, which included 76 women with an average age of 36 ± 11.9 years and 24 males with an average age of 31.54 ± 12.97 years. There was no significant difference between the age of the case and control groups in the male group, the female group and the total population (P: 0.09, 0.323, 0.089) (Table 1).

**Table 1:** comparing age and iron markers in patients with migraine and controls

		**Case**	**Control **	**Statistic **	**P **
Male	Number	24	24	-	-
	Age	31.54 ± 12.97	37.13 ± 8.93	1.737	.09
	Hb	14.82 ± 2.03	14.51 ± 2.03	-.519 ^a^	.606
	Ferritin	103.13 ± 65.95	134.54 ± 159.62	.891 ^a^	.38
	IDA	4 (16.7%)	7 (29.2%)	1.061 (1) ^b^	.303
Female	Number	76	76	-	-
	Age	36 ± 11.9	37.79 ± 10.28	.992	.323
	Hb	12.87 ± 1.4	13.8 ± 1.08	3.721	.0004
	Ferritin	43.09 ± 37.08	63.69 ± 39.06	2.814	.006
	IDA	17 (22.4%)	3 (3.9%)	11.285 (1)	.001
Total	Number	100	100	-	-
	Age	34.93 ± 12.25	37.63 ± 9.93	1.712	.089
	Hb	13.59 ± 1.9	13.97 ± 1.39	1.41	.161
	Ferritin	65.25 ± 57.23	80.69 ± 89.44	1.35	.178
	IDA	21 (21%)	10 (10%)	4.619 (1)	.032

Abbreviations: IDA: Iron Deficiency Anemia; (a) *t*-statistic; (b) χ^2^-statistic (df); (c) *P* ≤ .05 was considered as statistically significantly different

**Comparison of total hemoglobin in case and control groups**

**Figure 1:**
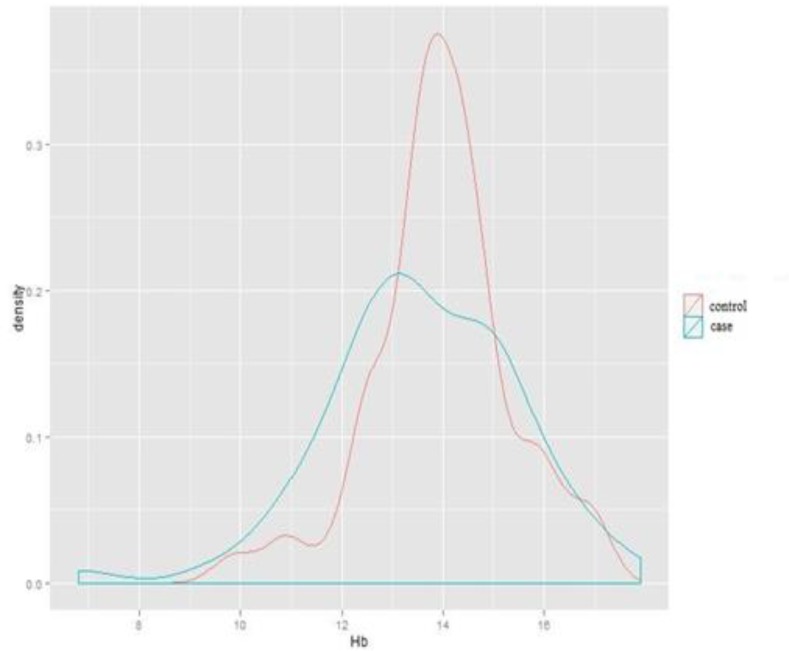
Dispersion and density of hemoglobin in case and control groups without gender segregation (Independent students’ T-test).

As indicated in Figure 1, there was no statistically significant difference in hemoglobin level between the subjects suffering from migraine and the subjects in the control group (P: 0.161); however, hemoglobin levels were found to be less distributed in the control group.

**[**Control mean ± SD: 13.97 ± 1.39, case mean ± SD: 13.59 ± 1.9 P: .161 t-statistic: 1.41]

**Comparison of the ferritin level in case and control groups**

**Figure 2:**
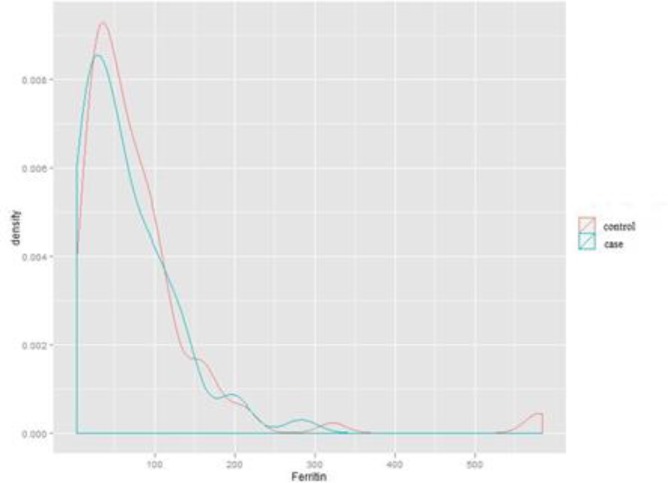
Dispersion and density of ferritin in case and control groups without gender segregation (Independent students’ T-test).

Based on Figure 2, there was no statistically significant difference in the level of ferritin between subjects with migraine and the subjects in the control group (P: 0.178), and the two similar diagrams to each group have a similar pattern.

**[**Control mean ± SD: 80.69 ± 89.44, case mean ± SD: 65.25 ± 57.23, P: .178, t-statistic: 1.35]

**Comparison of the prevalence of IDA in the study population in case and control groups**

**Figure 3:**
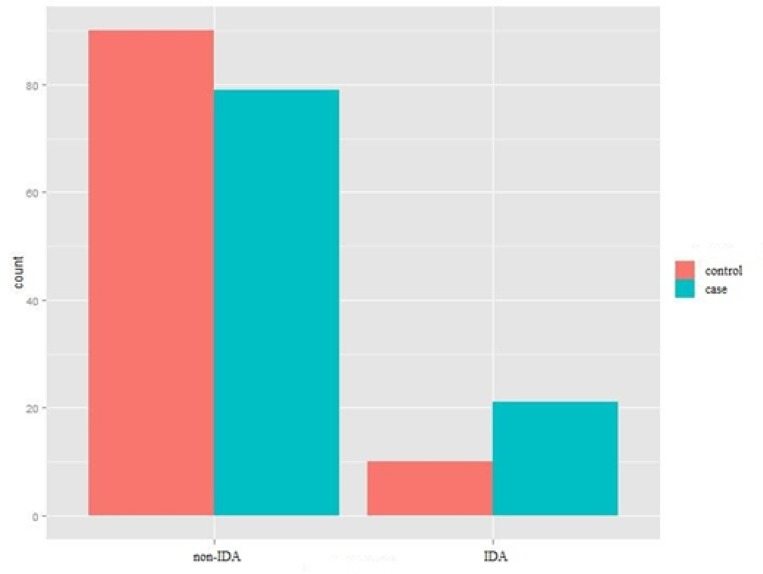
prevalence of IDA in the case and control groups without gender segregation (Chi-square test for independence).

As indicated in Figure 3, the relative prevalence of IDA in the migraine group (21%) was higher than the control group (10%), and no statistically significant difference was observed between the subjects suffering from migraine and the subjects in the control group ( P: 0.032).

[P: .032, χ^2^-statistic (df): 4.619 (1)]

**Comparison of hemoglobin level in male subjects in case and control groups**

**Figure 4:**
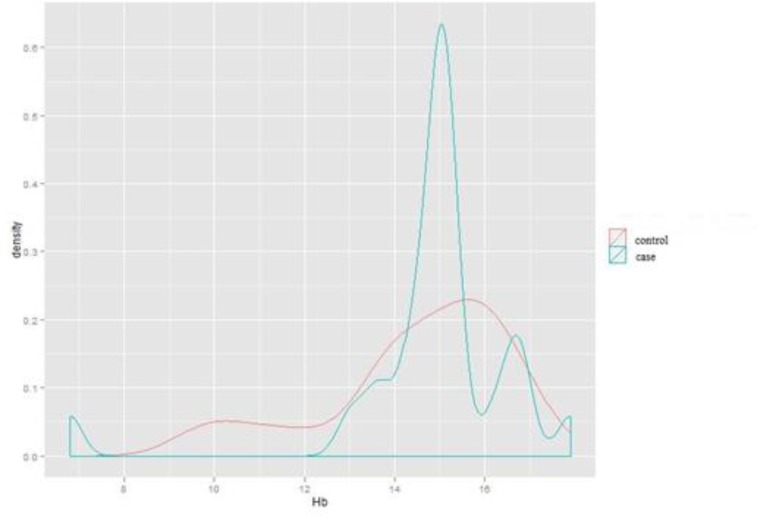
Dispersion and density of hemoglobin in the male case and control groups (Independent students’ T-test).

Figure 4 shows the distribution and density of hemoglobin in male subjects. As seen, there was no significant difference in the level of hemoglobin between the subjects suffering from migraine and the subjects in the control group (P: 0.606); however, it can be observed that hemoglobin levels are less frequent in the migraine group.

**[**Control mean ± SD: 14.51 ± 2.03 case mean ± SD: 14.82 ± 2.03, P: .606, t-statistic: -.519]

**Comparison of ferritin level in male subjects in case and control groups**

Descriptive drawing 5 reveals the dispersion and density of ferritin among male subjects in case and control groups. As seen, there was no significant difference in the level of ferritin between the subjects suffering from migraine and the subjects in the control group (P: 0.38).

**Figure 5:**
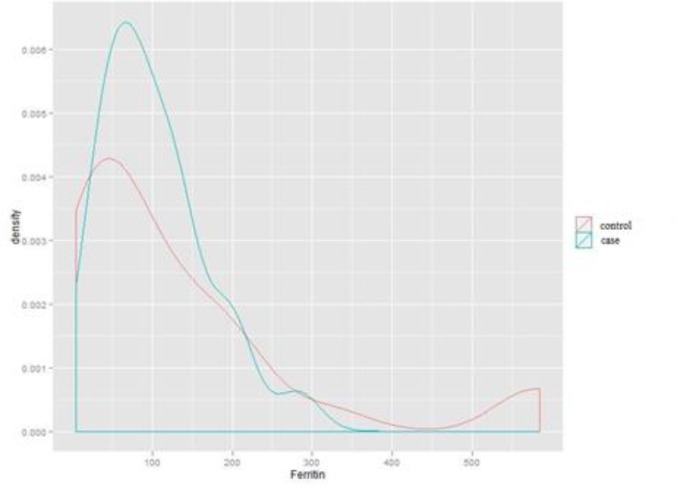
Dispersion and density of ferritin in the male case and control groups (Independent students’ T-test).

**[**Control mean ± SD: 134.52 ± 159.62, case mean ± SD: 103.13 ± 65.95 P: .38, t-statistic: .891]

**Prevalence of male subjects' IDA in case-control in male subjects**

Descriptive drawing 6 points to the prevalence of IDA in men. As can be seen, although the relative prevalence of IDA in the control group (29.2%) was higher than the migraine group (16.7%), there was no statistically significant difference between the subjects with migraine and the subjects in the control group (P: 0.303).

**Figure 6:**
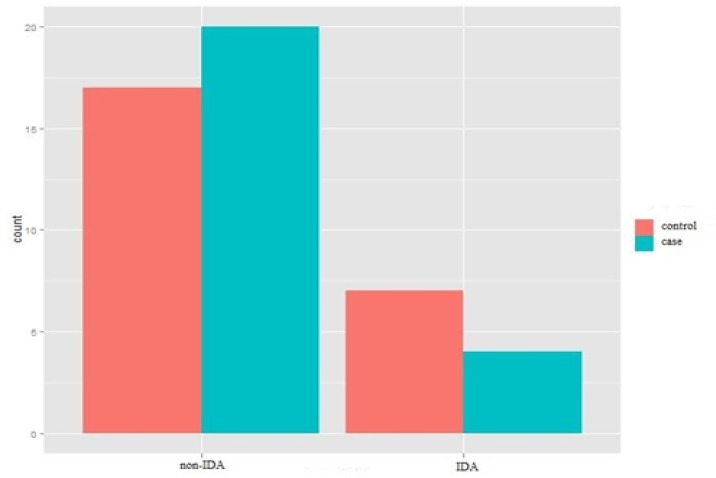
prevalence of IDA in the male case and control groups (Chi-square test for independence).

[P: .303, χ2-statistic (df): 1.061 (1)]

**Comparison of hemoglobin level in females in case and control groups**

Descriptive drawing 7 indicates the dispersion and density of hemoglobin among female subjects in case and control groups. As shown, there was a statistically significant difference in the level of hemoglobin between the subjects suffering from migraine and the subjects in the control group (P- value: 0.0004). Migraine sufferers showed higher level of hemoglobin compared to the control group.

**Figure 7:**
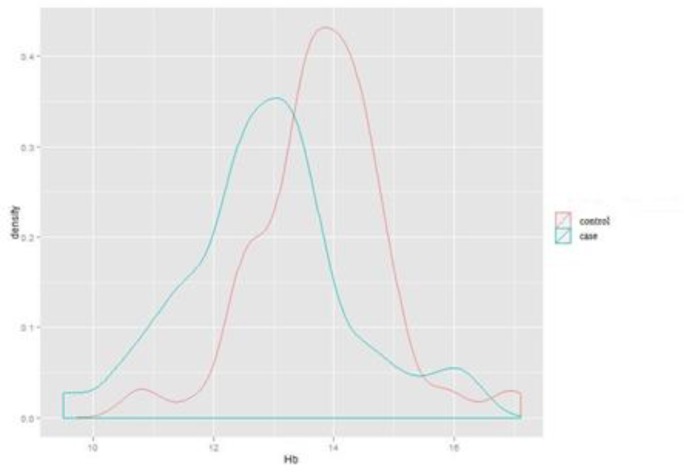
Dispersion and density of hemoglobin in the female case and control groups (Independent students’ T-test).

[Control mean ± SD: 13.8 ± 1.08, case mean ± SD: 12.87 ± 1.4, P: .0004, t-statistic: 3.721]

**Comparison of ferritin level in female subjects in case and control groups**

Descriptive drawing 8 shows the dispersion and density of ferritin among female subjects in case and control groups. As shown, there was a statistically significant difference in the level of ferritin between the subjects suffering from migraine and the subjects in the control group (P: 0.006).

**Figure 8:**
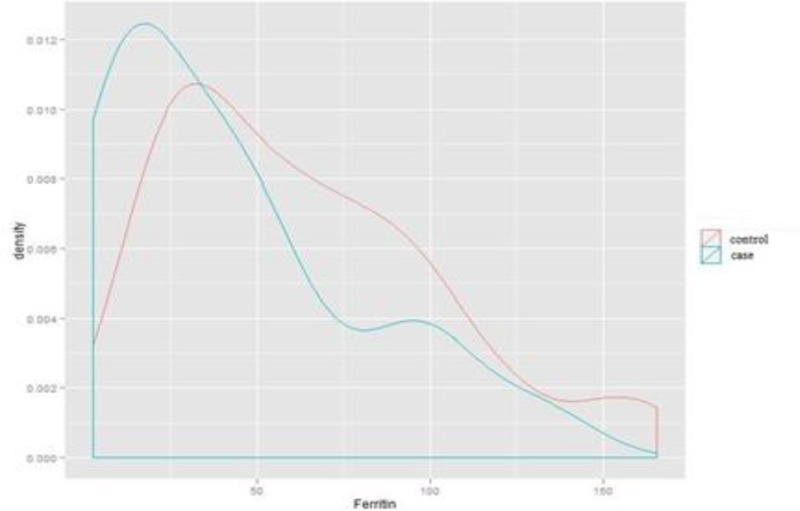
Dispersion and density of ferritin in the female case and control groups (Independent students’ T-test).

**[**Control mean ± SD: 63.69 ± 39.06, case mean ± SD: 43.09 ± 37.08 P: .006, t-statistic: 2.814]

**Prevalence of IDA in females in case and control groups**

Descriptive drawing 9 demonstrates the prevalence of iron-deficiency anemia among female subjects in case and control groups. As shown, the relative prevalence of iron-deficiency anemia in the migraine group (22%) was higher than the control group (3.9%), and a statistically significant difference was observed between the subjects suffering from migraine and the subjects in the control group (P: 0.001).

**Figure 9:**
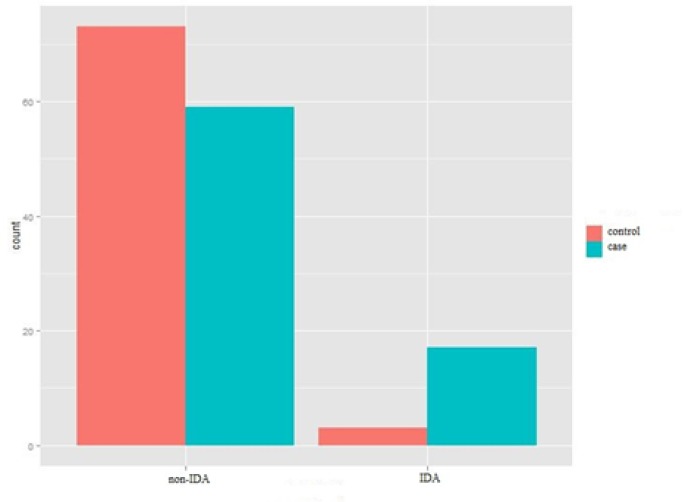
prevalence of IDA in thefe male case and control groups (Chi-square test for independence).

[P: .001, χ^2^-statistic (df): 11.285 (1)]

## Discussion

 For decades, migraine was considered as an episodic disease without long-term effects on the brain, but studies that have been conducted over the past three decades offer a different perspective. Accordingly, migraine can lead to subclinical brain lesions, posterior circulatory infarcts, inferotentorial hypertension lesions, and so on^8^. 

For the first time in 2001, based on magnetic resonance imaging (MRI), Welch et al. indicated that iron accumulation in the brain, especially in the periaqueductal gray (PAG) area, is associated with the duration of migrainous patients^9^. Eight years later, Kruit et al., consistent with previous findings, reported red nucleus (RN), globus pallidus (GP), and putamen iron accumulation in migraine patients^8^. It is noteworthy that Palm-Minders et al., in 2017, did not observe a difference between the migraine and the control group after conducting a follow-up study of the participants by Kruit et al. after 9 years, but noted that this result would probably be due to the increase in iron content in the brain tissue because of aging; therefore, age can be considered as one of the factors causing conflict^   10 ^.

In a possible explanation of the relationship between iron-deficiency anemia (IDA) and migraine, paying attention to the physiology of PAG and basal ganglia is essential. The amount of non-hem iron in areas such as GP, substantia nigra (SN), RN, and *cell*-*surface transferrin receptor* in PAG is in the highest level in comparison with other regions of the brain ^9^^,^^11^ , indicating the high metabolic activity of these areas (producing high values ​​of neurotransmitters). A possible explanation for the relationship between IDA and migraine is that by decreasing iron level of serum (decreasing hemoglobin and ferritin levels), on the surface of the transferrin receptors, upregulation occurs in the above-mentioned areas, and when the iron level is increased for any reason (e.g., receiving high doses of iron in girls and women), accumulation occurs. 

Another explanation is that considering the abundance of transferrin receptors, the activity of these areas due to migraine and iron absorption is high   9^, ^^12^.

Another possible mechanism is the altered dopaminergic function caused by IDA, since this change in dopaminergic function is considered as one of the triggers for migraine^   13 ^. Accordingly, recent findings indicate changes in the expression of three genes involved in nigrostriatal dopamine malfunction due to IDA^        14 ^. Therefore, the acceptable treatment of migraine obtained by D2 receptor agonists of Dopamine is justified^   15 ^. On the other hand, further studies are needed to prove or disprove this claim.

The decrease in estrogen levels before menstrual bleeding in a group of females suffering from iron deficiency may lead to migraine attacks ^16^^-^^19^ . This estrogen reduction causes hepcidine, a protein involved in iron metabolism synthesized in the liver^20^, and causes deficiency due to estrogen regulation in the expression of ferroportin^        21 ^. However, more studies are needed to investigate the interaction between IDA, estrogen, and migraine.

In a study conducted by Gur-Ozmen, the relationship between IDA and migraine was observed. This relationship was more significant among girls and women, as the relative prevalence of IDA in girls suffering from migraine was higher than that of men and boys. In addition, there was a significant relationship between migraine and hemoglobin level^   22 ^. All these results are consistent with the present study. However, unlike the current study, there was no significant relationship between migraine and ferritin levels in the study conducted by Gur-Ozmen. Also, in the current study, there was no significant difference among men in the case-control groups between IDA and migraine, which may reflect the fact that IDA is not common among men. Another explanation for this finding is the small sample size of the male population in our study. Future studies can provide a satisfactory response to these issues.

In addition to the above, several studies have suggested the relationship between IDA and migraine, or the least footprint of this possible relationship, including a number of case-controls and some cross-sectional studies. Pamuk et al. stated that IDA patients had high migraines, depression, and high stress, which can be indicative of an underlying relationship between IDA and diseases and central nervous system problems^2^. A similar study conducted by Keyvani et al. in Iran states that there is a significant relationship between IDA and migraine headache^   23 ^.

Additionally, the relationship between hemoglobin level and cognitive function in IDA patients^24^, improvement of linguistic and memory learning by iron supplementation in girls without iron- deficiency anemia^25^ and an increased frequency of the restless legs syndrome in IDA patients^26^ would be some evidence of central roles of iron.

Iron is necessary for monoamine oxidase enzyme (MAO) synthesis and decreasing MAO simultaneously in IDA and migraine and its increase after iron supplementation 6^, ^^30^^, ^^31^  suggests the effect of iron regimen on the severity and prevalence of migraine attacks in both IDA and non-IDA groups. This issue was supposed to be a link between IDA and migraine

## CONCLUSION

 The present study indicates the relationship between hemoglobin, ferritin, as well as IDA and migraine, especially in women and girls. Therefore, according to the results, treatment for iron- deficiency anemia or iron supplementation may be suggested as a suitable treatment or prevention method for patients suffering from both migraine and IDA at the same time. Further studies are still needed to provide a comprehensive response to the issues discussed.
